# Supplementary material for: Altered microRNA Transcriptome in Cultured Human Liver Cells upon Infection with Ebola Virus
Source: Int J Mol Sci. 2021 Apr 6;22(7):3792. doi: 10.3390/ijms22073792 (PMC8038836; doi:10.3390/ijms22073792)
Supplement: Supplementary file 1 [file ijms-22-03792-s001.zip › Supplementary_File/C_ GO_Analysis_Results/16-30nt_go_Mayinga-96h-Huh7_vs_Control-96h-Huh7_down.mature_mirna_targets/BP_Pvalue_tree.pdf]

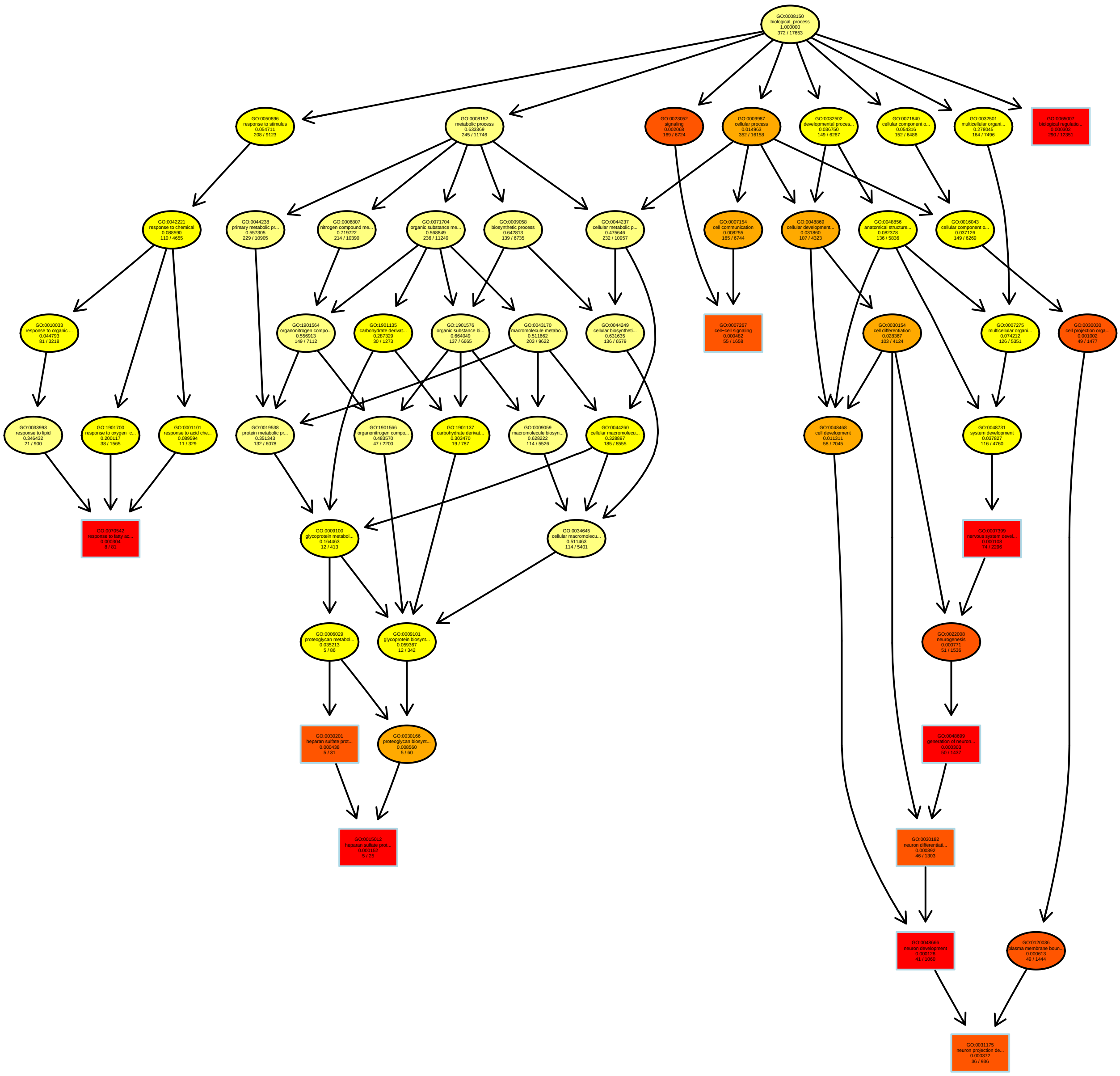

GO:0008150 biological_process 1.000000 372 / 17653
GO:0050896 response to stimulus 0.054711 208 / 9123
GO:0008152 metabolic process 0.633369 245 / 11746
GO:0023052 signaling 0.002068 169 / 6724
GO:0009987 cellular process 0.014963 352 / 16158
GO:0032502 developmental proces... 0.036750 149 / 6267
GO:0071840 cellular component o... 0.054316 152 / 6486
GO:0032501 multicellular organi... 0.278045 164 / 7496
GO:0065007 biological regulatio... 0.000302 290 / 12351
GO:0042221 response to chemical 0.088590 110 / 4655
GO:0044238 primary metabolic pr... 0.557305 229 / 10905
GO:0006807 nitrogen compound me... 0.719722 214 / 10390
GO:0071704 organic substance me... 0.568849 236 / 11249
GO:0009058 biosynthetic process 0.642813 139 / 6735
GO:0044237 cellular metabolic p... 0.475646 232 / 10957
GO:0007154 cell communication 0.008255 165 / 6744
GO:0048869 cellular development... 0.031860 107 / 4323
GO:0048856 anatomical structure... 0.082378 136 / 5836
GO:0016043 cellular component o... 0.037126 149 / 6269
GO:0010033 response to organic ... 0.044793 81 / 3218
GO:1901564 organonitrogen compo... 0.556913 149 / 7112
GO:1901135 carbohydrate derivat... 0.287329 30 / 1273
GO:1901576 organic substance bi... 0.664049 137 / 6665
GO:0043170 macromolecule metabo... 0.511662 203 / 9622
GO:0044249 cellular biosyntheti... 0.631635 136 / 6579
GO:0007267 cell−cell signaling 0.000482 55 / 1658
GO:0030154 cell differentiation 0.028367 103 / 4124
GO:0007275 multicellular organi... 0.074212 126 / 5351
GO:0030030 cell projection orga... 0.001002 49 / 1477
GO:0033993 response to lipid 0.346432 21 / 900
GO:1901700 response to oxygen−c... 0.200117 38 / 1565
GO:0001101 response to acid che... 0.089594 11 / 329
GO:0019538 protein metabolic pr... 0.351343 132 / 6078
GO:1901566 organonitrogen compo... 0.483570 47 / 2200
GO:1901137 carbohydrate derivat... 0.303470 19 / 787
GO:0009059 macromolecule biosyn... 0.628222 114 / 5526
GO:0044260 cellular macromolecu... 0.328897 185 / 8555
GO:0048468 cell development 0.011311 58 / 2045
GO:0048731 system development 0.037827 116 / 4760
GO:0070542 response to fatty ac... 0.000304 8 / 81
GO:0009100 glycoprotein metabol... 0.164463 12 / 413
GO:0034645 cellular macromolecu... 0.511463 114 / 5401
GO:0007399 nervous system devel... 0.000108 74 / 2296
GO:0006029 proteoglycan metabol... 0.035213 5 / 86
GO:0009101 glycoprotein biosynt... 0.059367 12 / 342
GO:0022008 neurogenesis 0.000771 51 / 1536
GO:0030201 heparan sulfate prot... 0.000438 5 / 31
GO:0030166 proteoglycan biosynt... 0.008560 5 / 60
GO:0048699 generation of neuron... 0.000303 50 / 1437
GO:0015012 heparan sulfate prot... 0.000152 5 / 25
GO:0030182 neuron differentiati... 0.000392 46 / 1303
GO:0048666 neuron development 0.000128 41 / 1060
GO:0120036 plasma membrane boun... 0.000613 49 / 1444
GO:0031175 neuron projection de... 0.000372 36 / 936
